# Research on fabric surface defect detection algorithm based on improved Yolo_v4

**DOI:** 10.1038/s41598-023-50671-7

**Published:** 2024-03-06

**Authors:** Yuanyuan Li, Liyuan Song, Yin Cai, Zhijun Fang, Ming Tang

**Affiliations:** Shanghai University of Engineering Science, Songjiang, Shanghai, 201620 China

**Keywords:** Electrical and electronic engineering, Mechanical engineering

## Abstract

In industry, the task of defect classification and defect localization is an important part of defect detection system. However, existing studies only focus on one task and it is difficult to ensure the accuracy of both tasks. This paper proposes a defect detection system based on improved Yolo_v4, which greatly improves the detection ability of minor defects. For K_Means algorithm clustering prianchors question with strong subjectivity, the paper proposes the Density Based Spatial Clustering of Applications with Noise (DBSCAN) algorithm to determine the number of Anchors. To solve the problem of low detection rate of small targets caused by insufficient reuse rate of low-level features in CSPDarknet53 feature extraction network, this paper proposes an ECA-DenseNet-BC-121 feature extraction network to improve it. And the Dual Channel Feature Enhancement (DCFE) module is proposed to improve the local information loss and gradient propagation obstruction caused by quad chain convolution in PANet networks to improve the robustness of the model. The experimental results on the fabric surface defect detection datasets show that the mAP of the improved Yolo_v4 is 98.97%, which is 7.67% higher than SSD, 3.75% higher than Faster_RCNN, 10.82% higher than Yolo_v4 tiny, and 5.35% higher than Yolo_v4, and the detection speed reaches 39.4 fps. It can meet the real-time monitoring needs of industrial sites.

## Introduction

In the modern textile industry, vision-based fabric surface inspection plays a crucial role in ensuring the quality of various textiles. Due to the problems of high labor intensity, low efficiency, and human error based on manual detection, the development of automated detection systems based on machine learning has attracted widespread attention in recent decades^1^. Since the end of the last century, various algorithms and techniques have been used to solve fabric inspection problems, including filter-based methods, feature-based methods, and learning-based methods^2,3^. While many of the proposed methods have shown impressive results for certain specific types of textures and defects. However, due to the complexity and difficulty of texturing analysis, fabric defect detection remains a challenging problem to be solved in the industry.

In general, fabric images can be seen as components of similar periodic textures that can be removed by manually designed filters to make it easier to separate retained defects. However, completely decoupling defects from the texture background is a rather difficult problem, and these problems stem from several factors, such as texture changes, lack of prior information about defects, and the complexity of certain types of texture patterns^4^. Based on the above reasons, a large number of research directions have shifted to feature extraction frameworks, essentially treating texture defects as decision problems in feature space, by extracting appropriate features from the original image and comparing them with features in a defect-free sample, defects can be identified as significant differences in feature space^5^. The key to this approach is how to design highly differentiated features to effectively distinguish defect areas from normal areas. In fact, this difference feature is highly dependent on the specific texture type already in place, but there are no clear rules to guide the selection of this feature^6^.

In recent years, the application of deep learning frameworks in fabric inspection and other visual fields has become increasingly popular and has received widespread attention^7^. Compared with traditional shallow learning methods, deep learning can learn from training samples to richer hierarchical features, and is widely used in the classification and detection of surface defects. The network model established by deep learning can automatically extract rich features from the training sample through the backpropagation algorithm under the guidance of the objective function, without the need to manually construct features based on experience, and truly realize end-to-end rapid defect detection^8^.

Deep learning models are usually a neural network structure that can automatically learn and extract the features of input data, which solves the complexity and uncertainty of manually extracted features in traditional machine vision. The deep network structure enables it to learn complex features, so a large number of researchers apply deep learning to detect product surface defects to improve the speed and accuracy of defect detection. RCNN network is the first deep learning technology to be applied to object detection tasks, but due to its high network complexity and cumbersome training process, it consumes a lot of computing and storage resources, and its practicability is poor ^9^. Therefore, the researchers further improved RCNN^10^, greatly reduced the complexity of the model by using feature sharing, simplified the training process of the network, and improved the real-time performance of the model. Faster RCNN^11^ is the first end-to-end detection model that shows significant improvements in real-time and accuracy compared to previous methods. In the early years, some studies generally applied Faster RCNN directly to industrial images, such as fabrics, PCB boards, steel surface damage detection, sanitary ceramics^12^ and polymer polarizers ^13^ defect detection, high-voltage line insulator ^14^ surface defect detection, etc., this method can achieve better detection results than the previous inspection method. Cheng ^2^ et al. applied Faster RCNN to damage detection of drainage system pipes to 83% mAP. In order to meet the application requirements of specific scenarios or to further improve the detection accuracy, many improved structures of Faster RCNN have been proposed successively. In literature^15^, in order to identify surface defects of freight vehicles, shallow, middle and deep features are simultaneously applied in defect identification, and the fully connected layer is replaced by a convolution layer to reduce the number of parameters. Compared with Faster RCNN, the proposed method can achieve a higher detection accuracy of 99.13%, and the number of model parameters is reduced by 17%.

Because the efficiency of the two-stage detection network cannot meet the industrial requirements in many scenarios, subsequent one-stage detection networks such as Yolo^16^, SSD^17^, and CornerNet^18^ are presented. The one-stage detection network regards defect classification and location as a unified regression task, and removes the step of candidate region extraction, which greatly improves the detection speed. When Suong et al.^19^ used Yolo_v2 network to detect the pit defects on the road surface, they recalculated the setting parameters of Anchor frame and hyperparameters such as raster size according to the shape distribution of defects and image resolution. In addition, the author deleted the original three convolutional layers of Yolo_v2, which greatly reduced the number of parameters. Zhang et al. ^20^ used Yolo_v3 network to detect bridge surface damage, and used batch re-standardization and Focal Loss to improve network performance. Yin et al. ^21^ used Yolo_v3 to detect the damage defects of sewage pipelines and obtained 85.37% mAP. In addition, Maeda et al.^22^ used SSD network to detect road surface defects. The authors compared the two backbone networks of Inception V2 and MobileNet and found that the detection accuracy of MobileNet is better than that of Inception V2, with the accuracy of identifying some types of defects reaching 99%. Moreover, the processing time of a single image using a mobile phone is less than 1.5 s, which realizes real-time detection. Compared with target objects in natural scenes, product defects in industry tend to be smaller and less distinctive. Therefore, the author combined multiple deep learning models into a joint model by means of cascade, so that the network could focus on the defect target with low proportion and improve generalization.

Based on the original Yolo object recognition architecture, Yolo_v4 algorithm has different degrees of optimization in the backbone network, activation function, loss function, model training and other aspects. Compared with the previous generations of algorithms, Yolo_v4 algorithm greatly improves the accuracy. It adopts the sliding window classification idea to predict classification and positioning under the same network architecture. At the application level, the current research is not very accurate in improving the accuracy and speed of fabric defect identification algorithm.

The purpose of this study is to solve the problem of error detection and leakage detection caused by traditional algorithms in the identification of defects under complex background conditions. Fabric defects in different backgrounds were taken as recognition objects, and the clustering, backbone feature extraction network and PANet network in Yolo_v4 target recognition algorithm were improved to improve the accuracy of the recognition algorithm and ensure the feature extraction speed of the algorithm.

In addition, based on the analysis of the advantages and disadvantages of the existing defect detection system, aiming at the problems of strong subjectivity of the clustering prior frame (Anchors) of the K_Means algorithm, low detection rate of small targets caused by insufficient reuse rate of low-level features by CSPDarknet53 feature extraction network, local information loss and obstruction of gradient propagation caused by five-time chain convolution in PANet network. We propose a defect detection system based on improved Yolo_v4, the contributions of this paper are as follows.

- Use the density based spatial clustering of applications with noise (DBSCAN) algorithm to determine the number of Anchors.
- Improve the ECA-DenseNet-BC-121 feature extraction network, and propose the dual channel feature enhancement (DCFE) module to improve the robustness of the model.
- In order to verify the feature mining ability of the algorithm for difficult samples, the paper designs a fusion detection experiment to verify the detection ability of the model for small defects.
- Use the proposed method to train and test on the DAGM 2007 dataset, the mAP of the proposed algorithm is 98.97%, and the detection speed reaches 39.4 fps, which can meet the real-time monitoring needs of industrial sites, show that the method based on Yolo_v4 is feasible.

The rest of this paper is organized as follows. Section “Proposed method” describes the technical details of our proposed method. The results and analysis of our experiments are presented in “Experimental results and analysis”. Finally, we conclude the paper in “Conclusion”.

## Proposed method

### Yolo_v4 network model

The basic idea of Yolo's algorithm is to divide the input image into S × S mesh graphs after a series of convolutional operations, and each grid will generate B bounding boxes. Each mesh is responsible for the detection of its lower-right corner area, and if the center point of the detected target falls in that area, the position of the target is detected by this grid and the confidence level of the bounding box is calculated. Since the features of small objects are easily lost after multiple convolutional compression of the image, the Yolo_v4 algorithm uses three feature maps of different scales (13 × 13, 26 × 26, 52 × 52) to predict targets at different scales. Large feature maps are used to detect large objects, and small feature maps are used to detect small objects. In Yolo_v4, cells of three scales are split into different numbers of cells, each of which predicts 3 bounding boxes, each bounding box is represented by (x, y, w, h, c) parameters, (x, y) represents the coordinates of the center point of the target relative to the upper-left corner of the cell in which it is located, (w, h) represents the ratio of the width and height of the target to the input image, and c represents the confidence. For C different categories of targets, each bounding box predicts the probability value of each target category, so each cell in the yolo v4 can predict 3 × (5 + C) probability values. On this basis, the bounding box with low confidence level is removed by setting the confidence threshold. Finally, the Yolo_v4 uses DIOU NMS to filter the remaining bounding boxes to obtain the final detection results. The bounding box confidence is calculated as follows:

$$Conf(obj) = P_{r} (Obj) \times IOU_{pre}^{truth}$$

1 $$IOU_{pre}^{truth} = \frac{{area\left( {B_{pre} \cap B_{truth} } \right)}}{{ \, area\left( {B_{pre} \cup B_{truth} } \right)}}$$

where *Conf*(*obj*) represents the confidence level of the detection target; *P*_*r*_(*obj*) indicates whether the center point of the detection target is in the prediction box, such as 1 in the prediction box, otherwise 0; $IOU_{pre}^{truth}$ Indicates the degree of coincidence between the real box and the prediction box; *B*_*pre*_ represents a prediction box; *B*_*truth*_ represents a real box; *area*(*B*_*pre*_ ∩ *B*_*truth*_) represents the area where the prediction box coincides with the real box; *area*(*B*_*pre*_ ∪ *B*_*truth*_) represents the area where the real box and the prediction box are unified.

The Yolo_v4 network structure is mainly composed of CSPDarknet53 feature extraction network, SPP module, PANet feature fusion module, and yolo head classifier.

**Figure 1:**
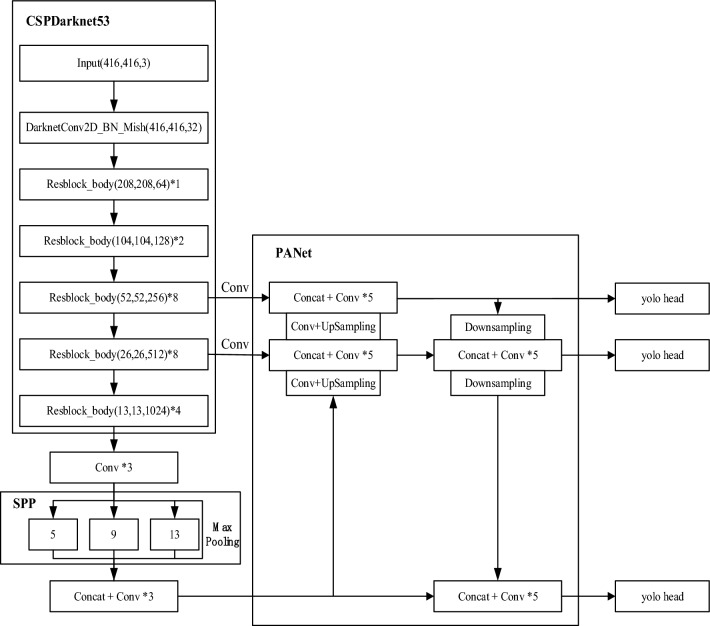
Yolo_v4 network structure diagram.

As shown in Fig. 1, first adjust the input image to the size of 416 × 416, and then input into the CSPDarknet53 backbone feature extraction network, and the feature maps of 3 different scales are obtained by 8-fold, 16-fold and 32-fold downsampling respectively, that is, the feature maps of 52 × 52, 26 × 26 and 13 × 13 shown in the figure. Among them, the 13 × 13 feature map output by the last layer of CSPDarknet53 was convolved three times by DarknetConv2D_BN_Leaky and then input to the SPP structure for processing.

The SPP module uses four different sizes (5 × 5, 9 × 9, 13 × 13, 13, 1 × 1) of the maximum pooling check input feature map for processing, SPP structure can effectively increase the receptive field of the feature map and isolate the most significant contextual features. The size of the feature map after SPP structure processing is unchanged, only the number of channels is increased.The feature diagrams of 52 × 52, 26 × 26 and 13 × 13 processed by SPP are then imported into the PANet module for processing. The PANet module is essentially an improved version of the feature pyramid structure. The difference is that the feature pyramid structure ends after the feature fusion is completed, which is sampled layer by layer from the bottom up, PANet also realizes feature extraction from top to bottom, and achieves the purpose of feature enhancement through repeated feature extraction and fusion.Finally, the feature maps of 52 × 52, 26 × 26, 13 × 13 processed by PANet module are input into three identical yolo head classifiers for classification and the detection results are obtained.

The activation function of Yolo_v4 has been changed from *LeakyRelu* in yolo_v3 to *Mish*. The advantage of the *Mish* activation function is that it is not completely truncated at x < 0, but rather allows a relatively small inflow of negative gradients to ensure the flow of information. In addition, the *Mish* activation function also guarantees smoothness at each point, which makes the gradient descent work better than *LeakyRelu*. The *Mish* function expression is as follows:

2 $$\, Mish \, = x \times \tanh \left( {\ln \left( {1 + e^{ \wedge } x} \right)} \right)$$

### Improved Yolo_v4 model

#### Improved clustering algorithm

##### Principles of the K-Means algorithm

K-Means is the most classic representative of the clustering algorithm, its basic idea is straightforward, and it is widely used. For a given data sample, K-Means will divide the sample data into K classes according to the distance between samples, so as to ensure that the intra-class distance is as close as possible and the inter-class distance is as far as possible.Set the various types (C1, C2, …, Ck), then the objective function of K-Means can be expressed as a squared error E, the expression of which is as follows:

3 $$E = \sum\limits_{i = 1}^{k} {\sum\limits_{{x \in C_{i} }} {\left\| {x - \mu_{i} } \right\|_{2}^{2} } }$$

where x is the data sample and μi is the mean vector (centroid) of category Ci, the formula is:

4 $$\mu_{i} = \frac{1}{{\left| {C_{i} } \right|}}\sum\limits_{{x \in C_{i} }} x$$

**Figure 2:**
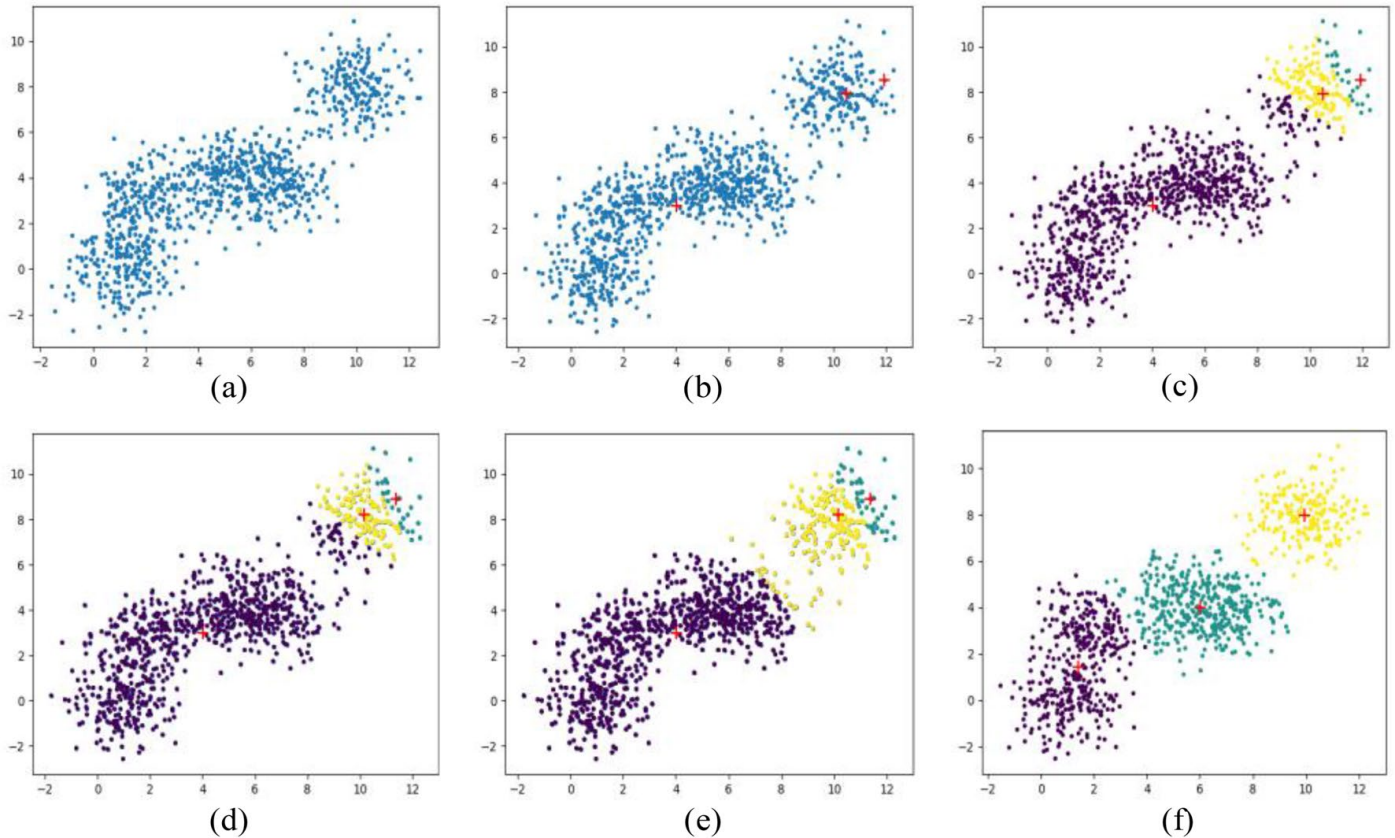
The process of K-Means three classification iterative.

Figure 2 shows the iterative process of K-Means algorithm to achieve three classifications. Figure 2a shows the initial distribution of data samples, and set k = 3. According to the classification requirements of the sample data, three centroids are randomly selected in Fig. 2b, and then the distance between all data points in the sample to the two centroids is calculated and compared, and those close to the centroid are marked as this class. As shown in Fig. 2c, by calculating the distances of all samples from the three types of centroids, the categories of all data points after the first iteration can be obtained.The new centroid is then re-found for each of the current data samples, and Fig. 2d show the updated centroid location. Repeat the procedure described in Fig. 2c again to obtain all updated data categories, as shown in Fig. 2e. Finally, three clustering results as shown in Fig. 2f are obtained.

The K-Means clustering process is summarized below:

(a) First randomly select K objects from a given data sample as the initial clustering center; (b) Then calculate the distance of each sample point to the center of the cluster for categorization; (c) Update the cluster center again according to the divided sample category; (d) Repeat the process (b) and (c) until the maximum number of iterations is reached.

##### Principles of DBSCAN clustering algorithm

DBSCAN^23^ (Density-Based Spatial Clustering of Application with Noise) is a representative density clustering-based clustering algorithm, which defines clusters as the largest set of densely connected points, can divide areas with sufficient density into clusters, and can find arbitrarily shaped clusters in noisy data.

The DBSCAN algorithm has six basic concepts: *ε-neighborhood*, core object, boundary point, outlier point, direct density reach, and density reach.

*ε*-neighborhood: Neighborhood within the radius r of a given object. K-Means algorithm is based on distance calculation, but in DBSCAN, the most core parameter is the radius, which will have a great impact on the results; Core object: An object is a core object if its ε-neighborhood contains at least m data points; Boundary points: Boundary points are not core points, but fall in the neighborhood of some core point, that is, the boundary position in the data set; Outliers: Other data points that are neither core points nor boundary points are outliers; Direct density reachability: Given a set D of objects, if *p* is in an *ε*-neighborhood of *q* and *q* is a core point, then object *p* is directly density reachability from object *q*; Density reachable: If there exists a chain of objects *p*_*1*_*, p*_*2*_*,*… *p*_*n*_*, p*_*1*_ = *q, p*_*n*_ = *p*, object *p*_*i*_ belongs to D, and *p*_*i*+*1*_ is density-reachable from *p*_*i*_ with respect to *ε* and* m*, then object *p* is density-reachable from object *q* with respect to *ε* and *m*.

The pseudocode for the DBSCAN algorithm flow is as follows:
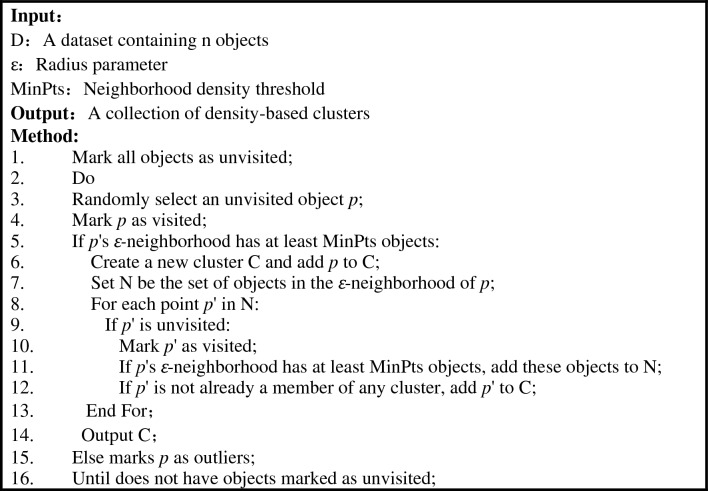


Yolo_v4 uses K-Means clustering algorithm to obtain Anchor Boxes. In K-Means algorithm, the choice of K value is usually given subjectively, and in many cases, it is not known in advance how many categories a given data set should be clustered. In addition, K-Means adopts Euclidean distance and Manhattan distance as similarity indicators, which also has great problems. However, the disadvantage of DBSCAN algorithm is that it is very difficult to select the radius, and the results obtained by different radii often have huge differences.

In view of the above problems, this paper combines the advantages of the two algorithms to carry out Anchor Boxes clustering, and the specific steps are as follows:

1. Firstly, use DBSCAN algorithm to analyze the outliers in the data sample, eliminate the interference of outliers on the clustering results, and determine the K value adaptively for the K-Means clustering algorithm.
2. The K-Means clustering algorithm is further used to determine the center of the cluster, and the distance formula is as follows:
  5 $$D(X,Y) = 1 - {\text{IOU}} (X,Y)$$
  where X represents the real box; Y represents the cluster box; IOU(X,Y) represents the intersection ratio between the center box of the target and the cluster box.

**Figure 3:**
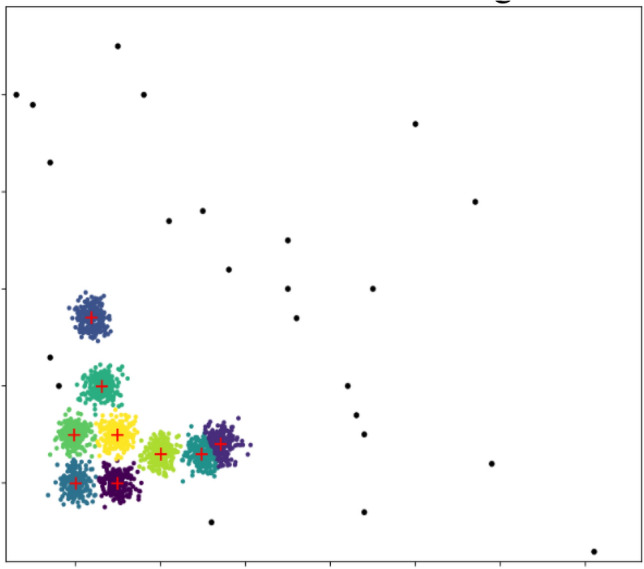
Clustering results.

As shown in Fig. 3, the maximum value of K in K-Means algorithm determined by DBSCAN algorithm is 9, and the black points in the figure are outliers. Obviously, the combination of DBSCAN algorithm and K-Means algorithm for clustering can ignore the influence of outliers, dig out better clustering centers, and make the algorithm more robust.

Since most of the data sets selected in this paper are small defect samples, although it is generally believed that the more Anchor Boxes are, the higher the prediction accuracy will be, the computation will be multiplied and a large amount of redundancy will be brought, which will increase the burden of subsequent non-maximum suppression (NMS) algorithm. Therefore, this paper determines the most appropriate number of Anchor Boxes by comparing the size of Avg IOU (mean intersection and union ratio) when K takes different values. Avg IOU is calculated using the following formula:

6 $$AVG\_IOU = \arg \max \frac{{\sum\nolimits_{i}^{k} {\sum\nolimits_{j}^{{n_{k} }} {IOU(X,Y)} } }}{n}$$

where *n* represents the total number of data samples; *n*_*k*_ represents the number of samples at the kth cluster center; *k* represents the number of clustering centers.

**Figure 4:**
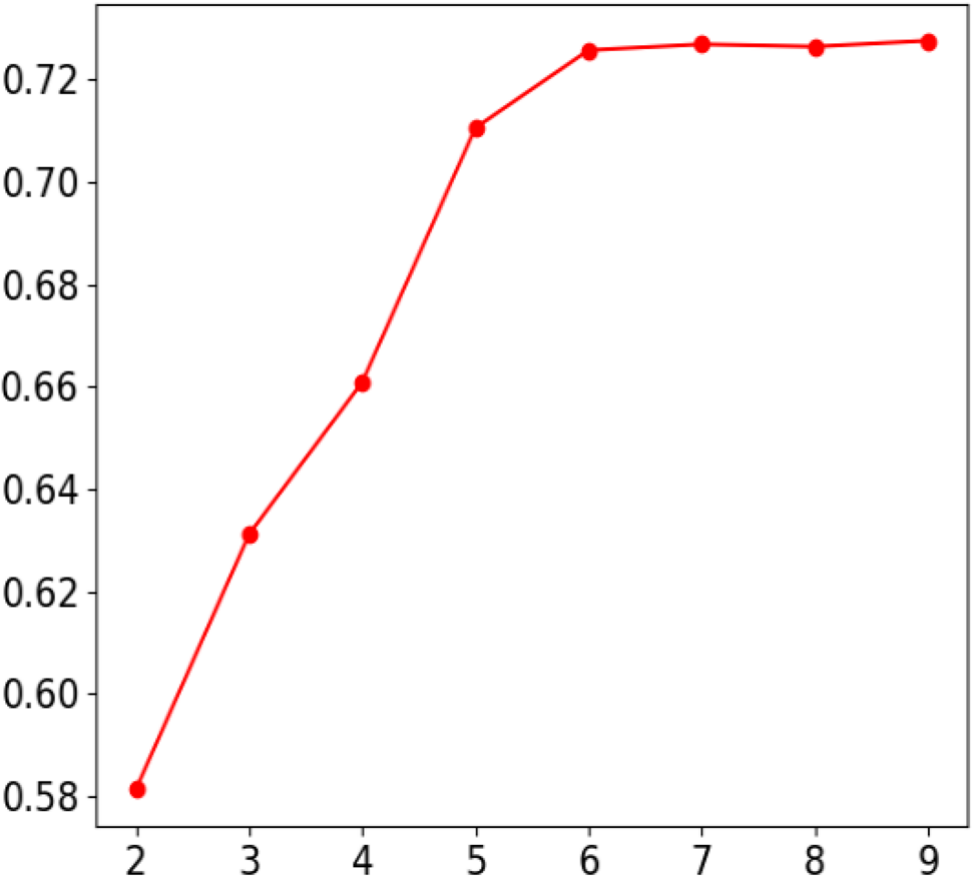
Avg IOU accuracy curve.

Set k = 2–9 to perform cluster analysis on the real box in the sample of the dataset, and the results are shown in Fig. 4. Obviously, with the continuous increase of k value, although Avg IOU value increases continuously, the final curve tends to be stable, and the numerical difference between the curve at k = 9 and k = 6 is not obvious. Considering the calculation amount and the fact that most of the data sets in this paper are small defects, six clustering centers are selected as Anchor Boxes in this paper. Respectively (10, 18), (16, 32), (30, 30), (76, 24), (102,112), (104,47).

### Improvements to the backbone feature extraction network

**Figure 5:**
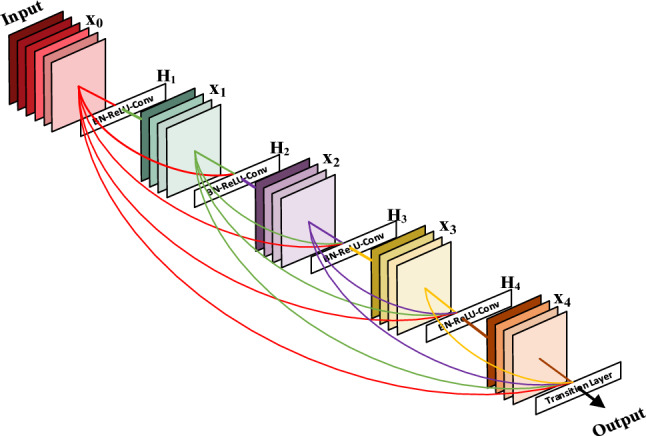
DenseNet network structure.

Figure 5 shows the DenseNet^24^ network structure. DenseNet network is a kind of three-dimensional cross-layer dense connection. For l-layer convolutional neural networks, DenseNet networks have l(l + 1)/2 layer connections, that is, the input of each layer comes from the output of all previous layers.

The output of a DenseNet network at the *l* layer is:

7 $$x_{l} = H_{l} \left( {\left[ {x_{0} ,x_{1} , \ldots ,x_{l - 1} } \right]} \right)$$

where *[x*_*0*_*,x*_*1*_*,…,x*_*l−1*_*]* is a stitching of feature maps generated at layers *0,1,…,l − 1*. *H*_*l*_(·) is a nonlinear conversion function. DenseNet, as another convolutional neural network with a deeper number of layers, has the following advantages:

- The way of cross-layer connection enhances the transfer of features;
- The cross-level structure has a certain regular effect, making the network easier to train;
- Alleviates the problem of gradient disappearance and performance degradation;
- The amount of parameters is reduced to a certain extent.

Table 1 shows the network architecture of various versions of DenseNet. In the table, k is the growth rate, representing the number of feature maps output by each Dense Block module. Compared with the CSPDarknet53 network, the DenseNet network has fewer output feature maps, but it can bring better performance. The main reason is that the cross-layer structure of DenseNet network can fully integrate the features of the lower layer and the higher layer to ensure the effective transmission of information. Dense Block module on each Layer includes 1 × 1 convolution and 3 × 3 convolution. 1 × 1 convolution is called Bottleneck Layer here. The input feature map information can be aggregated through 1 × 1 convolution, so as to reduce the number of feature maps and reduce the computational burden for the subsequent 3 × 3 convolution. At the same time, 1 × 1 convolution and average pool operation are used for further parameter compression between each two layers of Dense Block. This structure is called Transition Layer. In addition, a compression parameter σ(0 < σ < 1) is introduced in this layer, which means that the output feature map will be reduced to how many times the original one. It is usually 0.5. There are many versions of DenseNet, among which DenseNet-BC represents a structure with Bottleneck Layer and Transition Layer.

**Table 1:** DenseNet architecture.

Layers	Output size	DenseNet-BC-121 (k = 32)	DenseNet-BC-169 (k = 32)	DenseNet-BC-201 (k = 32)	DenseNet-BC-264 (k = 48)
Convolution	112 × 112	7 × 7 conv, stride 2			
pooling	56 × 56	3 × 3 max pool, stride 2			
Dense block (1)	56 × 56	$$\left[ {\begin{array}{*{20}l} {1 \times 1{\text{ conv }}} \hfill \\ {3 \times 3{\text{ conv }}} \hfill \\ \end{array} } \right] \times 6$$	$$\left[ {\begin{array}{*{20}l} {1 \times 1{\text{ conv }}} \hfill \\ {3 \times 3{\text{ conv }}} \hfill \\ \end{array} } \right] \times 6$$	$$\left[ {\begin{array}{*{20}l} {1 \times 1{\text{ conv }}} \hfill \\ {3 \times 3{\text{ conv }}} \hfill \\ \end{array} } \right] \times 6$$	$$\left[ {\begin{array}{*{20}l} {1 \times 1{\text{ conv }}} \hfill \\ {3 \times 3{\text{ conv }}} \hfill \\ \end{array} } \right] \times 6$$
Transition layer(1)	56 × 56	1 × 1 conv			
	28 × 28	2 × 2 average pool, stride 2			
Dense block (2)	28 × 28	$$\left[ {\begin{array}{*{20}l} {1 \times 1{\text{ conv }}} \hfill \\ {3 \times 3{\text{ conv }}} \hfill \\ \end{array} } \right] \times 12$$	$$\left[ {\begin{array}{*{20}l} {1 \times 1{\text{ conv }}} \hfill \\ {3 \times 3{\text{ conv }}} \hfill \\ \end{array} } \right] \times 12$$	$$\left[ {\begin{array}{*{20}l} {1 \times 1{\text{ conv }}} \hfill \\ {3 \times 3{\text{ conv }}} \hfill \\ \end{array} } \right] \times 12$$	$$\left[ {\begin{array}{*{20}l} {1 \times 1{\text{ conv }}} \hfill \\ {3 \times 3{\text{ conv }}} \hfill \\ \end{array} } \right] \times 12$$
Transition layer(2)	28 × 28	1 × 1 conv			
	14 × 14	2 × 2 average pool, stride 2			
Dense block (3)	14 × 14	$$\left[ {\begin{array}{*{20}l} {1 \times 1{\text{ conv }}} \hfill \\ {3 \times 3{\text{ conv }}} \hfill \\ \end{array} } \right] \times 24$$	$$\left[ {\begin{array}{*{20}l} {1 \times 1{\text{ conv }}} \hfill \\ {3 \times 3{\text{ conv }}} \hfill \\ \end{array} } \right] \times 32$$	$$\left[ {\begin{array}{*{20}l} {1 \times 1{\text{ conv }}} \hfill \\ {3 \times 3{\text{ conv }}} \hfill \\ \end{array} } \right] \times 48$$	$$\left[ {\begin{array}{*{20}l} {1 \times 1{\text{ conv }}} \hfill \\ {3 \times 3{\text{ conv }}} \hfill \\ \end{array} } \right] \times 64$$
Transition layer (3)	14 × 14	1 × 1 conv			
	7 × 7	2 × 2 average pool, stride 2			
Dense block (4)	7 × 7	$$\left[ {\begin{array}{*{20}l} {1 \times 1{\text{ conv }}} \hfill \\ {3 \times 3{\text{ conv }}} \hfill \\ \end{array} } \right] \times 16$$	$$\left[ {\begin{array}{*{20}l} {1 \times 1{\text{ conv }}} \hfill \\ {3 \times 3{\text{ conv }}} \hfill \\ \end{array} } \right] \times 32$$	$$\left[ {\begin{array}{*{20}l} {1 \times 1{\text{ conv }}} \hfill \\ {3 \times 3{\text{ conv }}} \hfill \\ \end{array} } \right] \times 32$$	$$\left[ {\begin{array}{*{20}l} {1 \times 1{\text{ conv }}} \hfill \\ {3 \times 3{\text{ conv }}} \hfill \\ \end{array} } \right] \times 48$$
Classification layer	1 × 1	7 × 7 global average pool			
		1000D fully-connected, softmax			

**Figure 6:**
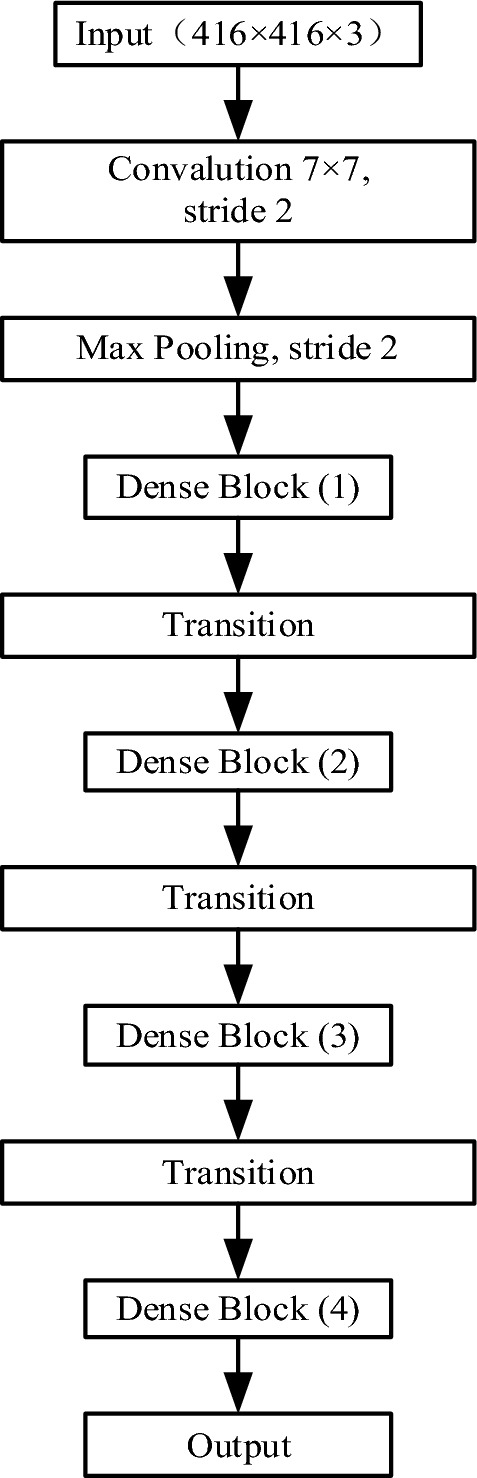
DenseNet-BC-121 network structure.

Considering the small sample size of the dataset used in this paper, in order to prevent problems such as overfitting caused by the network being too large, this paper uses the DenseNet-BC-121 structure to replace the original yolo-v4 network of CSPDarknet53 feature extraction network. Figure 6 shows the DenseNet-BC-121 network structure. Firstly, 64 convolution kernels with the size of 7 × 7 are used to downsample the input image to reduce the image dimension. Then use maximum pooling to continue to downsample the feature map; Then we use 4 Dense blocks and 3 Transition layers to continuously extract features from the feature map. Finally, the prediction result is output.

**Figure 7:**
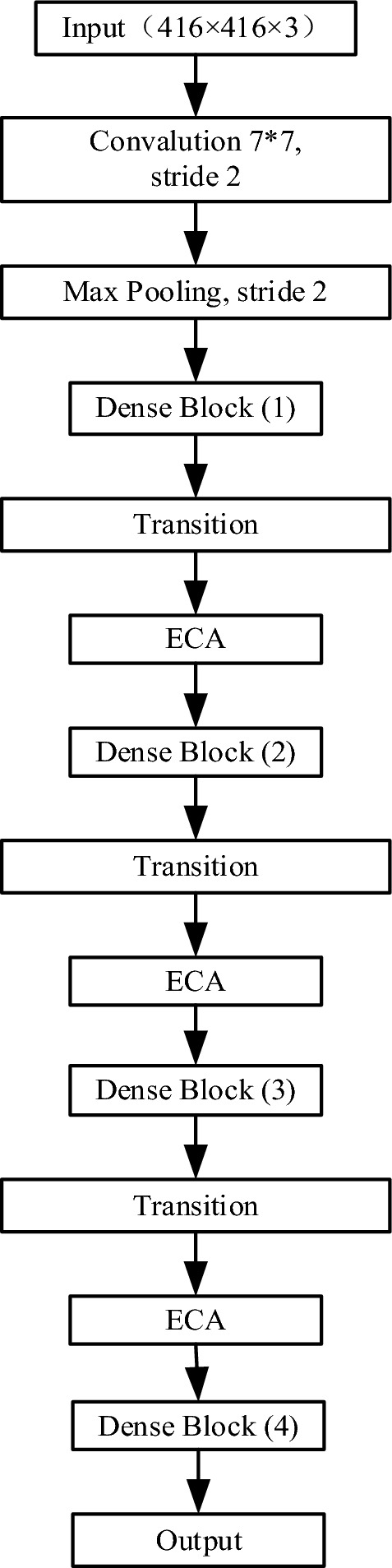
ECA-DenseNet-BC-121 network structure.

In order to make the DenseNet-BC-121 feature extraction network have certain feature screening ability, which can enhance the effective feature information of each layer and suppress the invalid feature information, this paper introduces the attention mechanism ECA into the structure to obtain the feature extraction network ECA-DenseNet-BC-121, the structure of which is shown in Fig. 7. ECA^25^ first performs global averaging pooling of the input feature map to obtain a set of one-dimensional feature vectors equal to the number of channels of the input feature map, and then performs a nonlinear transformation of the obtained vector through a full connection operation, so that it can be adaptively optimized according to the objective function of the model, and finally obtains a set of attention weights by the sigmoid function, and multiplies it with the input feature map to obtain the attention feature map.

#### Improvements to PANet network

**Figure 8:**
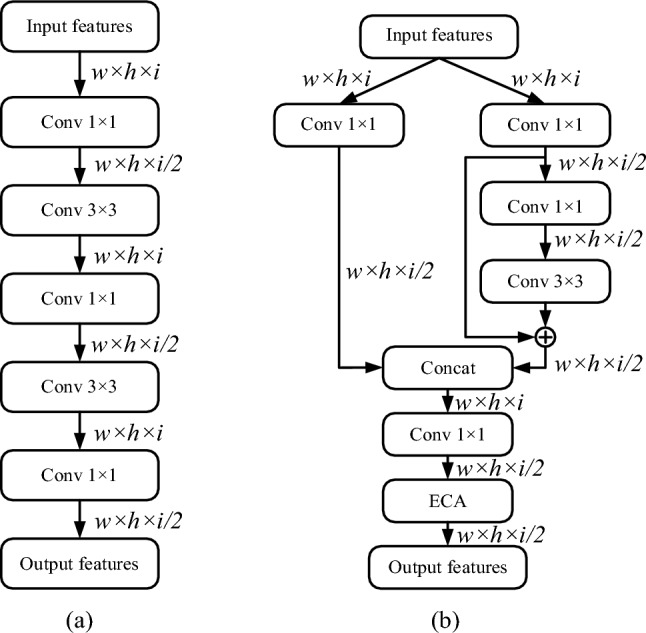
(**a**) Five times of convolution before improvement. (**b**) Five times of convolution after improvement.

In PANet network, after concat feature fusion, five times of convolution is performed to extract features repeatedly. Its structure is shown in Fig. 8a. There are three main disadvantages of this structure: (1) The chain feature extraction structure is not conducive to the transmission of information, easy to cause the loss of local information; (2) The chain structure is not conducive to the back propagation of the gradient, which is easy to cause the network training stagnation, unable to learn deeper features, and thus trapped in the local optimal; (3) There is no feature screening mechanism in this structure, which can not enhance the network capability by strengthening the effective features.

In view of the above shortcomings, this paper proposes its improved structure Dual channel feature enhancement (DCFE) module. As shown in Fig. 8b, in the DCFE module, the input feature map is divided into left and right parts for feature extraction respectively, and the left part only performs 1 × 1 convolution once. The number of channels is reduced to half of the input while the channel feature is aggregated. In the right part, a 1 × 1 convolution is first performed to adjust the number of channels to half of the input, and then feature fusion is performed through a residual module. The feature maps on the left and right sides are superimposed by concat operation, and then the number of channels is adjusted by a 1 × 1 convolution. Finally, the attention mechanism (ECA) module is used to screen the features.

Compared with the improved five-fold basic convolution module, the DCFE module has four main advantages: (1) The dual-channel structure can improve the reusability of features to a certain extent; (2) The addition of right residual structure can effectively prevent the disappearance of the gradient and enhance the learning ability of the network; (3) The two-channel structure itself also constitutes residual effect, and this two-level residual mechanism further improves the network learning ability; (4) Finally, the attention mechanism introduced can screen the features after all the features are fused, so as to suppress the invalid features and strengthen the effective features.

**Figure 9:**
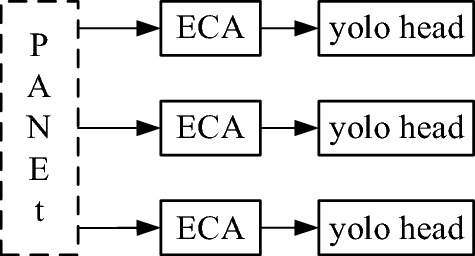
Importing attention mechanism on the detection end.

At the same time, in order to improve the accuracy of fabric defect detection and classification and ensure that the model pays more attention to the location information while predicting defect categories, the ECA module is also introduced between PANet and yolo_head for feature screening, as shown in Fig. 9.

**Figure 10:**
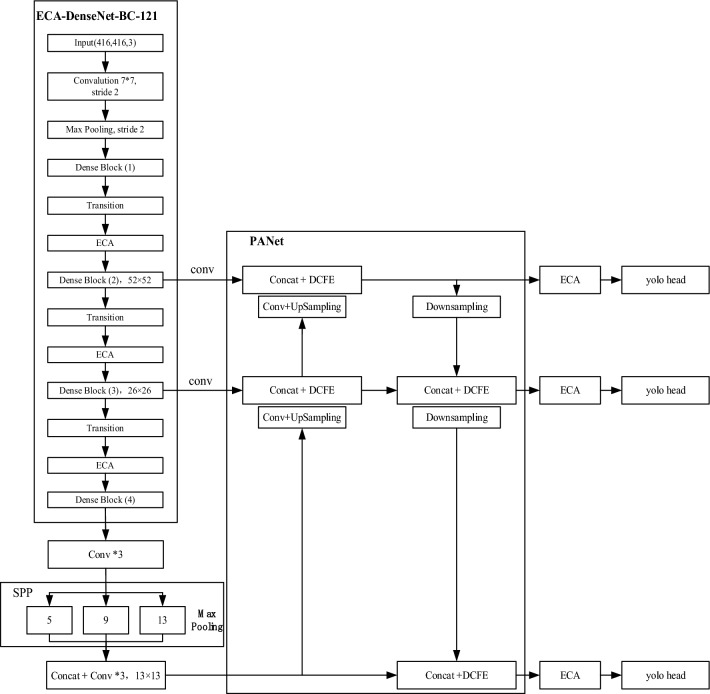
Improving the overall framework Yolo_v4.

Figure 10 shows the overall structure of the improved Yolo_v4 network. The image to be detected is preprocessed and adjusted to the size of 416 × 416, and then input into the ECA-DenseNet-BC-121 network for feature extraction. The parameters of each layer are shown in Table 2. The obtained feature maps with sizes of 52 × 52, 26 × 26 and 13 × 13 were input to the improved PANet network for feature enhancement and fusion, and the 13 × 13 feature maps were input to the SPP network for receptive field enhancement after three convolution (1 × 1, 3 × 3, 1 × 1). The size of the feature map obtained by PANet network processing is 52 × 52 × 90, 26 × 26 × 90, 13 × 13 × 90, where 90 = 6 × (5 + 10) (6 is the number of anchors obtained by clustering; 5 to determine the center point coordinates, length, width and confidence parameters required for a detection box; 10 is the number of categories); After feature screening by ECA module, the attention feature information is obtained. Finally, execute the regression operation through yolo_head to get the final detection result.

**Table 2:** Architecture of ECA-DenseNet-BC-121.

Layers	Output size	ECA-DenseNet-BC-121(k = 32)
Convolution	208 × 208	7 × 7 conv, stride 2
Pooling	104 × 104	3 × 3 max pool, stride 2
Dense block (1)	104 × 104	$$\left[ {\begin{array}{*{20}l} {1 \times 1{\text{ conv }}} \hfill \\ {3 \times 3{\text{ conv }}} \hfill \\ \end{array} } \right] \times 6$$
Transition layer (1)	104 × 104	1 × 1 conv
	52 × 52	2 × 2 average pool, stride 2
ECA(1)	52 × 52	Global average pool, fc
Dense block (2)	52 × 52	$$\left[ {\begin{array}{*{20}l} {1 \times 1{\text{ conv }}} \hfill \\ {3 \times 3{\text{ conv }}} \hfill \\ \end{array} } \right] \times 12$$
Transition layer (2)	52 × 52	1 × 1 conv
	26 × 26	2 × 2 average pool, stride 2
ECA(2)	26 × 26	Global average pool, fc
Dense block (3)	26 × 26	$$\left[ {\begin{array}{*{20}l} {1 \times 1{\text{ conv }}} \hfill \\ {3 \times 3{\text{ conv }}} \hfill \\ \end{array} } \right] \times 24$$
Transition layer (3)	26 × 26	1 × 1 conv
	13 × 13	2 × 2 average pool, stride 2
ECA (3)	13 × 13	Global average pool, fc
Dense block (4)	13 × 13	$$\left[ {\begin{array}{*{20}l} {1 \times 1{\text{ conv }}} \hfill \\ {3 \times 3{\text{ conv }}} \hfill \\ \end{array} } \right] \times 16$$

The conv operations involved in the improved Yolo_v4 structure in this paper are composed of three parts: conv = convolution + batch standardization + Mish activation.

#### Loss function

The loss function is a measure of the consistency between the predicted value and the real value of the model output. The detection results of the algorithm proposed in this paper include the location, prediction category, confidence and other information of each prediction box. The constructed loss function must be able to evaluate all the above detection results and calculate the position error, category error and confidence error of the prediction results. Therefore, the loss function of the algorithm proposed in this paper consists of three parts, the formula is as follows:

8 $$loss = loss_{loc \, } + loss_{cla \, } + loss_{conf \, }$$

where $loss_{loc \, }$ represents position loss, $loss_{cla \, }$ represents category error, and $loss_{conf \, }$ represents confidence error.

The position loss is calculated using the $CIOU$ function, which fully considers the distance, overlap rate, scale and penalty term between the target and the anchor, so that the prediction box is closer to the real box, and there will be no oscillation and divergence problems in the training process like $IOU$, and secondly, the $IOU$ is the concept of ratio and is not sensitive to the scale of the target object. The formula for $CIOU$ is as follows:

9 $$CIOU = IOU - \frac{{\rho^{2} \left( {b,b^{gt} } \right)}}{{c^{2} }} - \alpha v$$

where the $IOU$ represents the ratio of the prediction box and the real box; *b* represents the center point coordinate of the prediction box, and $b^{gt}$ represents the center point coordinate of the real box; $\rho^{2} \left( {b,b^{gt} } \right)$ represents the Euclidean distance between the center point of the prediction box and the real box; c represents the diagonal distance of the smallest closure area that can contain both the prediction box and the real box. *α* and *v* are calculated as follows:

10 $$\alpha = \frac{v}{1 - IOU + v}$$

11 $$v = \frac{4}{{\pi^{2} }}\left( {\arctan \frac{{w^{gt} }}{{h^{gt} }} - \arctan \frac{w}{h}} \right)^{2}$$

where *w*^*gt*^,* h*^*gt*^ represents the width and height of the real box, and *w*, *h* represents the width and height of the prediction box. So *the loss*_*loc*_ formula is:

12 $$loss_{loc \, } = 1 - CIOU$$

The *loss*_*cla*_ formula for category error is as follows:

13 $$loss_{cla} = \sum\limits_{i = 0}^{{S^{2} }} {\prod\limits_{i}^{obj} {\sum\limits_{c \in cla} {\left( {p_{i} (c) - \hat{p}_{i} (c)} \right)^{2} } } }$$

where $\prod\limits_{i}^{obj} {}$ is used to determine whether there is a target object in the ith grid point; if there is, it is 1; if not, it is 0. *S* represents that the feature map is divided into* S* grid points. $\prod\limits_{i}^{obj} {}$ represents the classification score of the predicted category *c* of the forecast box. $\hat{p}_{i} (c)$ represents the true value of the category to which the forecast box belongs.

The confidence error *loss*_*conf*_ formula is as follows:

14 $$loss_{conf \, } = \sum\limits_{i = 0}^{{S^{2} }} {\sum\limits_{j = 0}^{B} {\prod\limits_{ij}^{obj \, } {\left( {C_{i} - \hat{C}_{i} } \right)^{2} } } } + \lambda_{noobj \, } \sum\limits_{i = 0}^{{S^{2} }} {\sum\limits_{j = 0}^{B} {\prod\limits_{ij}^{obj \, } {\left( {C_{i} - \hat{C}_{i} } \right)^{2} } } }$$

where $\prod\limits_{i}^{obj} {}$ is used to determine whether the *j(j* = *0,…,B)* prediction box in grid *i (i* = *0,…,S*^*2*^*)* is responsible for the detection of this target; $\lambda_{noobj \, }$ represents the weight coefficient of an object without a target in the mesh; $C_{i}$ is the confidence score; $\hat{C}_{i}$ is the intersection of the prediction box and the real box.

#### Transfer learning

Transfer learning can realize deep mining of data set features, extract domain knowledge and apply it to other similar fields. Transfer learning is highly adaptive. Compared with the traditional non-cross learning model, transfer learning has three main advantages: (1) It allows the training set and test set to follow different distributions; (2) Low requirements for data volume; (3) Model migration can also be realized in different fields.

Set *P* as the source task, *Q* as the target task, *D*_*P*_ and *D*_*Q*_ as the source domain and target domain, then they can be expressed as:

15 $$\left\{ {\begin{array}{*{20}l} {D_{P} = \left\{ {X_{P} ,P\left( {X_{P} } \right)} \right\}} \hfill \\ {D_{Q} = \left\{ {X_{Q} ,P\left( {X_{Q} } \right)} \right\}} \hfill \\ \end{array} } \right.$$

where *X* represents the eigenvector space in the domain, and *P(.)* denotes the corresponding probability distribution function.

Set *Y*_*p*_ and *Y*_*q*_ be label vector Spaces of source domain and target domain respectively, *f*_*p*_ and *f*_*q*_ be mapping functions of source domain and target domain respectively, then the tasks *T*_*p*_ and *T*_*q*_ in source domain and target domain can be described as follows:

16 $$\left\{ {\begin{array}{*{20}l} {T_{p} = \left\{ {Y_{p} ,f_{p} } \right\}} \hfill \\ {T_{q} = \left\{ {Y_{q} ,f_{q} } \right\}} \hfill \\ \end{array} } \right.$$

Assuming that the source domain and the target domain have the same feature distribution, but the label distribution is inconsistent, the goal of transfer learning is to minimize the error of the mapping function *f*_*q*_:* X*_*q*_* → X*_*p*_ on *D*_*q*_, and to satisfy *X*_*q*_ = *X*_*p*_, *Y*_*q*_ = *Y*_*p*_, and *P(Y*_*q*_*|X*_*q*_*) ≠ P(Y*_*p*_*|X*_*p*_*)*.

Due to the small number of samples in the fabric defect data set selected in this paper, it is easy to overfit the model if the model is directly trained from the beginning. Transfer learning can directly take the model parameters trained in domain A as the initial parameters of model training in domain B, and model B can get an excellent model only by fine-tuning the parameters on this basis. In general, domain A has an abundance of labeled data samples, while domain B has a scarcity of data samples. There are many kinds of defects in the fabric defect data set, but many kinds are not common and the number is small, which is difficult to meet the demand of deep learning for large data volume.

Therefore, firstly, the ECA-DenseNet-BC-121 network is trained on Pascal VOC dataset to obtain the pre-trained feature extraction network. Then, the fully connected layer of the pre-trained model is removed and transferred to the fabric defect detection model as a feature extractor. Then the subsequent structures such as SPP, PANet and yolo head were added to form the fabric defect detection model. Finally, the fine-tuning technique is used to fine-tune the model. The basic steps of fine-tuning are as follows: (1) Freeze all layers of the pre-trained network; (2) Train the added network layer; (3) Part of the thawing pre-training network layer; (4) Jointly train the thawed pre-trained network layer and the added network layer. In this paper, a total of 100 iterations were carried out in the fine-tuning process, and the first 50 iterations were frozen pre-training network process. The initial learning rate was 0.001, and Batch_size was set to 8. The last 50 iterations are the joint training process, the initial learning rate is 0.0001, and the Batch_size is set to 4. In order to prevent network jitter caused by excessive learning rate in the training process, the loss value of the model will be monitored during the training process. If the loss value of the model does not decrease in five consecutive iterations, the learning rate will be adjusted to 1/10 of the original one.

### Consent to participate

All members of this article agree to participate.

## Experimental results and analysis

### Experimental environment and experimental data

In order to verify the effectiveness of the method proposed in this paper, this paper builds an experimental platform under Win10 system, using Pytorch as the deep learning framework and python as the programming language. The CPU is Intel(R) Core(TM) i5-9600 k CPU@3.70 Hz, and the GPU is Nvidia RTX2060S.

**Figure 11:**
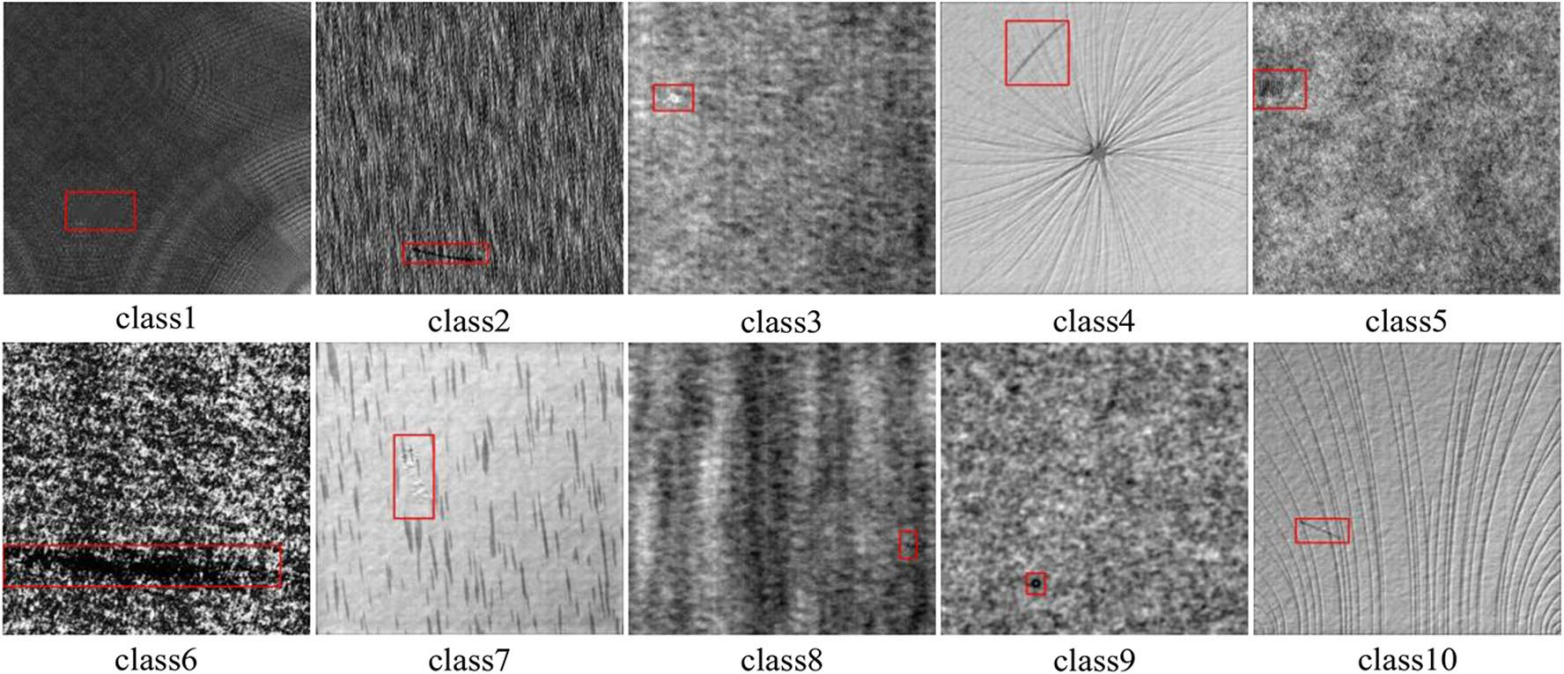
Ten types of defects in the DAGM 2007 dataset.

**Figure 12:**
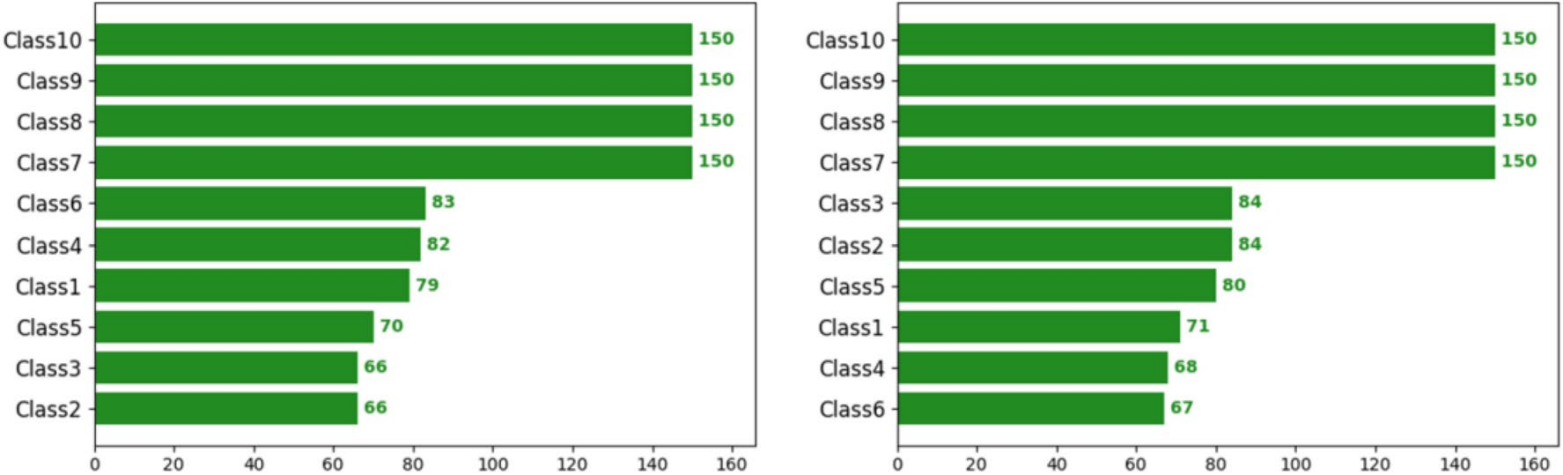
The number of samples of each type in the training set is on the left and the number of samples in the test set is on the right.

The dataset used in this paper is that of the open-source DAGM 2007 in Germany, with ten classes of texture defects, as shown in Fig. 11. The data set divides each class of data sample into training set and test set, most of which are defect-free samples. This paper selects all defect samples and makes them into a standard defect detection data set, including 1046 sample data and 10 sample data in the training set. The number of defect samples of each class is shown in Fig. 12.

The experiments in this section use multi-category mean accuracy (mAP) as an evaluation index to evaluate model performance.

### Model training

Considering that the training set has only 1046 pictures and a small sample size, in order to ensure the rigor of the experiment, the 30% cross-validation experiment is used to train the data set. In order to ensure sufficient training samples, 104 images are randomly selected as the validation set in each fold cross-validation experiment, and the remaining pictures are used as training subsets, and the basic process is as follows:

Training Set D, then divide the training set into three equal parts: D1 D2 D3,the training strategy is shown in the following Table 3:

**Table 3:** The training strategy.

	Training set			Validation set
First fold cross validation	D1	D2	Remaining images in D3	Randomly select 104 images in D3
Second fold cross validation	D2	D3	Remaining images in D1	Randomly select 104 images in D1
Third fold cross validation	D1	D3	Remaining images in D2	Randomly select 104 images in D2

**Figure 13:**
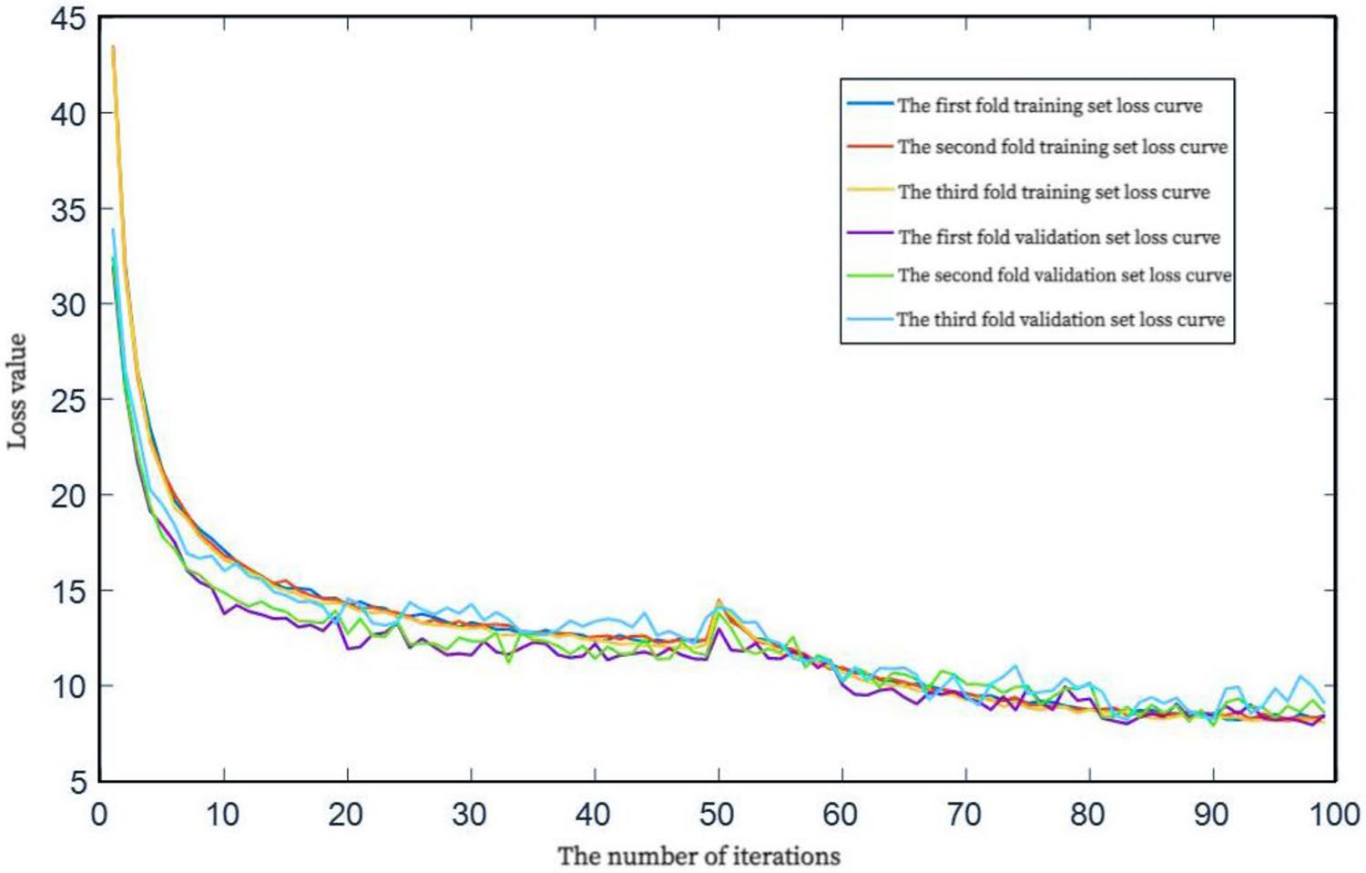
Loss curve.

The loss curve in the training process is shown in Fig. 13. It can be seen from the figure that in the first 50 times of transfer learning training, the loss value of both the training set and the validation set shows an obvious downward trend, and in the last 50 times of fine-tuning, the loss value steadily decreases and finally tends to be stable. During the whole training process, the decreasing trend of the training set loss and the validation set loss is basically the same, and there is no large oscillation, which indicates that the model design is reasonable and the fitting performance is good.

### Evaluation of defect detection results

Tables 4 and 5 show the detection results of the proposed algorithm on two datasets, where Base is the Yolo_v4 model. Base_A represents an improved clustering algorithm; Base_B represents the ECA-DenseNet-BC-121 feature extraction network; Base_C represents the DCFE module, and the performance indicator mAP@0.5 (the confidence threshold is set to 0.5) is used to compare the models.

**Table 4:** mAP on the strip surface defect detection datasets.

Model	mAP (%)	AP (%)					
		Sc	PAs	Rs	In	Ps	Cr
Base	61.46	86.17	78.65	61.65	57.10	54.81	30.42
Base_A	74.71	83.10	92.59	62.63	80.16	83.63	46.15
Base_AB	79.01	88.01	93.44	73.90	85.70	86.11	46.91
Base_ABC (ours)	**84.84**	**96.71**	**94.21**	**86.42**	**85.93**	**89.18**	**56.62**

Significant values are in bold.

**Table 5:** mAP on the fabric surface defect detection datasets.

Model	mAP (%)	AP (%)									
		C1	C2	C3	C4	C5	C6	C7	C8	C9	Cl0
Base	93.62	95.39	99.46	81.20	93.69	88.39	87.75	95.34	95.73	**100.0**	99.33
Base_A	94.82	91.24	95.34	91.17	99.01	98.48	83.13	95.50	94.40	**100.0**	**100.0**
Base_AB	97.19	95.12	98.95	94.11	99.19	99.24	92.03	97.47	95.85	**100.0**	**100.0**
Base_ABC (ours)	**98.97**	**95.61**	**100.0**	**98.62**	**99.97**	**99.44**	**99.61**	**98.79**	**97.70**	**100.0**	**100.0**

Significant values are in bold.

The detection accuracy mAP of the proposed method on the two datasets was 84.84% and 98.97%, respectively. Compared with the Base model, the accuracy is improved by 23.38% and 5.35%, respectively, and the performance is significantly improved. In order to compare the effects of A, B and C improvement strategies on model performance, four sets of control models are set up in this paper: Base, Base_A, Base_AB and Base_ABC to test the improvement effect. It can be seen from the table that mAP on both datasets shows an upward trend, and the results show that the three improvement strategies A, B and C have a promoting effect on the performance improvement of the model. For a single category of defect detection, due to the obvious difference between the various types of defects of the two datasets, the improved strategy in this paper also has a decrease in accuracy in individual defect types, for example, on the Scraches type defects of the strip datasets, the accuracy loss of the Base_A model compared with the Base model is 3.07%. This also shows that the three improvement strategies proposed in this model still have some room for optimization in the face of different types of defects.

Tables 6 and 7 show the F1-score evaluation results of the improved model on the two datasets, from which it can be seen that the proposed model is better than the Faster RCNN and Base models in most defect types, and only a certain degree of performance degradation occurs on the Rolled-in_scale defect types of the strip dataset, which basically achieves the expected detection effect. In addition, it can be seen from the data in the table that the F1-score performance improvement of the three improvement strategies proposed in this paper is positively superimposed on the two datasets, indicating the effectiveness of the improvement strategies.

**Table 6:** Comparison of F1-score results on strip surface defect detection datasets.

Model	F1-score					
	Sc	PAs	Rs	In	Ps	Cr
Faster RCNN	0.93	0.92	**0.87**	0.82	0.76	0.55
Base	0.89	0.86	0.68	0.74	0.78	0.45
Base_A	0.89	0.88	0.77	0.75	0.80	0.47
Base_AB	0.92	0.86	0.80	0.78	0.84	0.54
Base_ABC (ours)	**0.94**	**0.93**	0.85	**0.85**	**0.87**	**0.61**

Significant values are in bold.

**Table 7:** Comparison of F1-score results on fabric surface defect detection datasets.

Model	F1-score									
	C1	C2	C3	C4	C5	C6	C7	C8	C9	Cl0
Faster RCNN	**0.97**	0.99	0.93	0.95	0.98	0.94	0.96	0.94	**1.0**	**1.0**
Base	0.81	0.84	0.76	0.92	0.86	0.65	0.94	0.87	**1.0**	0.93
Base_A	0.85	0.92	0.85	0.96	0.94	0.79	0.94	0.92	**1.0**	**1.0**
Base_AB	0.96	0.96	0.90	0.97	**0.99**	0.87	0.96	0.94	**1.0**	**1.0**
Base_ABC (ours)	**0.97**	**1.0**	**0.98**	**0.99**	**0.99**	**0.98**	**0.97**	**0.97**	**1.0**	**1.0**

Significant values are in bold.

### Detection performance comparison

In this paper, the performance index mAP@0.5(confidence threshold is set as 0.5) is used to compare the improved Yolo_v4 algorithm with the mainstream deep learning models such as SSD, Faster_RCNN, Yolo_v4 tiny and Yolo_v4 on the test set of DAGM 2007 dataset. Table 6 lists the comparison results.

As can be seen from the Tables 8 and 9, the performance of the proposed algorithm is higher than that of the other four algorithms. In mAP on the fabric surface defect detection datasets, the proposed algorithm is higher than that of the other algorithms, and the average accuracy (AP) of a single class is higher than that of all the other algorithms except Class5. The mAP of the proposed algorithm is 98.97%, which is 7.67%, 3.75%, 10.82% and 5.35% higher than SSD, Faster_RCNN, Yolo_v4 tiny and Yolo_v4, respectively. In mAP on the strip surface defect detection datasets, the proposed algorithm is higher than that of the other algorithms. The mAP of the proposed algorithm is 80.86%, which is 5.01%, 3.65%, 1.51% and19.4% higher than SSD, Faster_RCNN, Yolo_v4 tiny and Yolo_v4, respectively.

**Table 8:** mAP on the fabric surface defect detection datasets.

Model	mAP (%)	AP (%)									
		Class 1	Class 2	Class 3	Class 4	Class 5	Class 6	Class 7	Class 8	Class 9	Class 10
SSD	91.30	92.32	91.49	87.20	90.59	88.85	91.92	92.40	89.06	93.71	95.52
Faster_RCNN	95.22	94.49	99.96	90.13	96.21	90.74	93.23	95.40	94.07	99.25	98.78
Yolo_v4 tiny	88.15	89.30	92.39	83.23	91.59	80.10	88.05	90.29	82.54	89.29	94.77
Yolo_v4	93.62	95.39	99.46	81.20	93.69	88.39	87.75	95.34	95.73	100.0	99.33
Ours	**98.97**	**95.61**	**100.0**	**98.62**	**99.97**	**99.44**	**99.61**	**98.79**	**97.70**	**100.0**	**100.0**

Significant values are in bold.

**Table 9:** mAP on the strip surface defect detection datasets.

Model	mAP (%)	AP (%)					
		Sc	PAs	Rs	In	Ps	Cr
SSD	75.85	77.01	91.38	85.93	72.54	78.26	49.99
Faster_RCNN	77.21	93.28	91.34	78.79	77.09	**79.47**	43.26
Yolo_v4 tiny	79.35	93.25	92.79	87.31	75.39	76.66	50.71
Yolo_v4	61.46	86.17	78.65	61.65	57.10	54.81	30.42
Ours	**80.86**	**93.66**	**93.90**	**89.72**	**77.84**	74.43	**55.61**

Significant values are in bold.

Table 10 shows the comparison of detection speed of each model. Although Faster RCNN algorithm can also provide good detection accuracy, the proposed algorithm is 4.1 times faster in detection speed and has better detection accuracy. As a lightweight detection model, Yolo_v4 tiny's detection speed is the fastest among all algorithms, but the decrease in the number of parameters also brings the loss of accuracy. The accuracy of this algorithm is the lowest among all comparison algorithms, which is difficult to meet the requirements of high-precision detection in industrial scenes. From the comparison experiment, it can be seen that the proposed algorithm can achieve a good balance in precision and speed.

**Table 10:** Comparison of detection speeds by model.

Model	SSD	Faster_RCNN	Yolo_v4 tiny	Yolo_v4	Our algorithm
Frame rate (fps)	35.3	9.5	64.2	41.5	39.4

### Single defect detection

In order to fully demonstrate the detection ability of the algorithm proposed in this chapter for small defects, each defect category in the strip datasets and the fabric datasets is stitched together by four similar pictures, compared with directly entering an original picture for detection, using this stitched picture as input network is equivalent to reducing the image resolution by 4 times, and the defects in the original picture will also be directly reduced by 4 times, which greatly increases the difficulty of detection, which also tests the generalization ability and feature mining ability of the algorithm.

**Figure 14:**
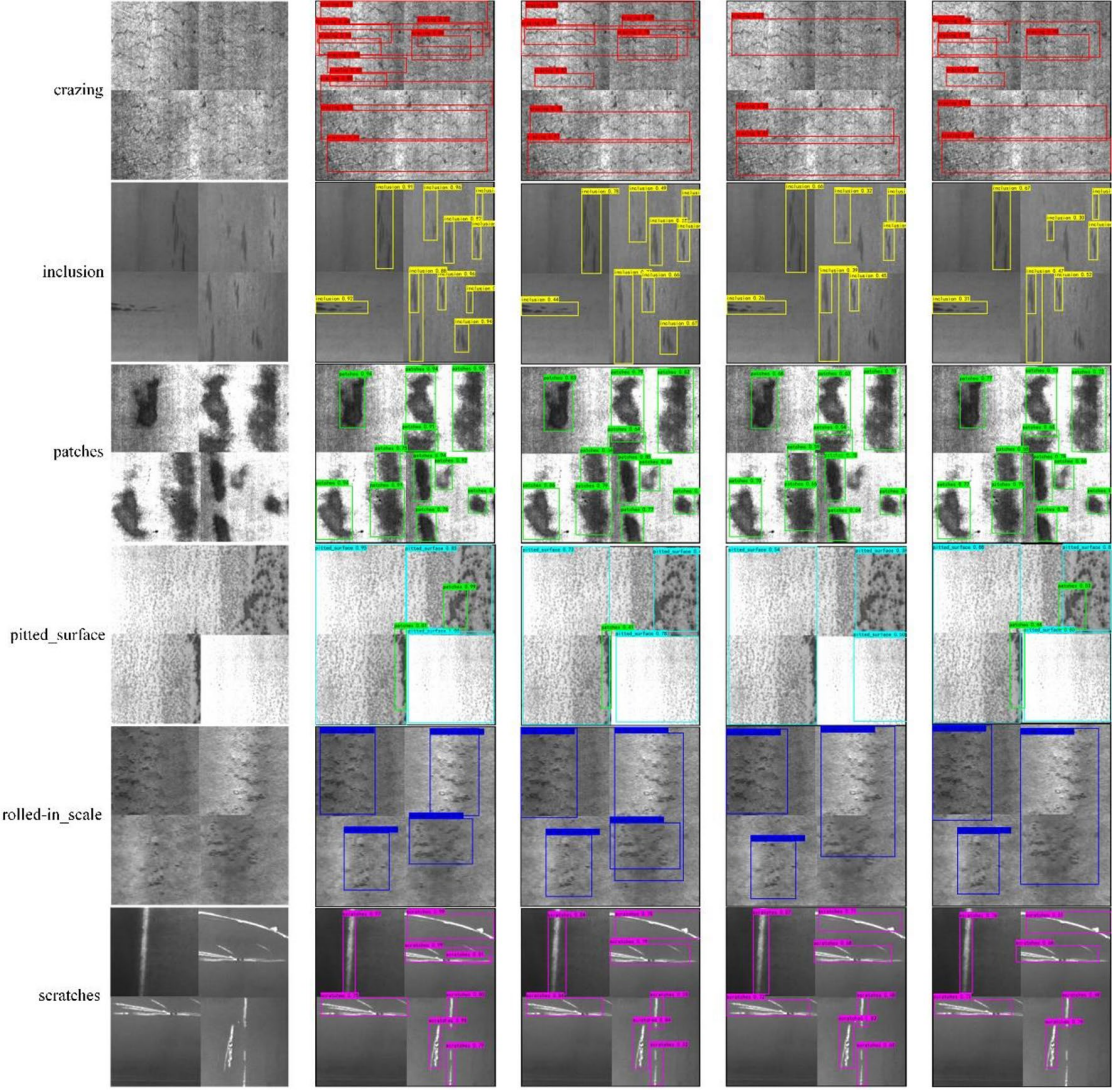
Comparison chart of single defect detection effect of strip datasets.

Figure 14 shows the comparison chart of single defect detection effect of strip datasets, from left to right, the original image, the proposed algorithm, Faster_RCNN, Yolo_v4 algorithms, and SSD. For the crazing defect categories that are difficult to detect in the strip datasets, the results are more detailed, and the algorithm in this paper can detect more non-obvious defect information than the other three algorithms. For the inclusion defect category, the average confidence of the algorithm in this paper reaches more than 0.9, while the comparative algorithms have missed detection to varying degrees, and the detection confidence is generally lower than 0.5. For the rolled_in-scale defect category, the proposed algorithm can detect more accurate position information, while the comparison algorithm has the situation of detection frame overlap and detection position deviation. Experiments show that the algorithm proposed in this paper can ensure higher confidence and more accurate position information for various defects in the strip datasets.

**Figure 15:**
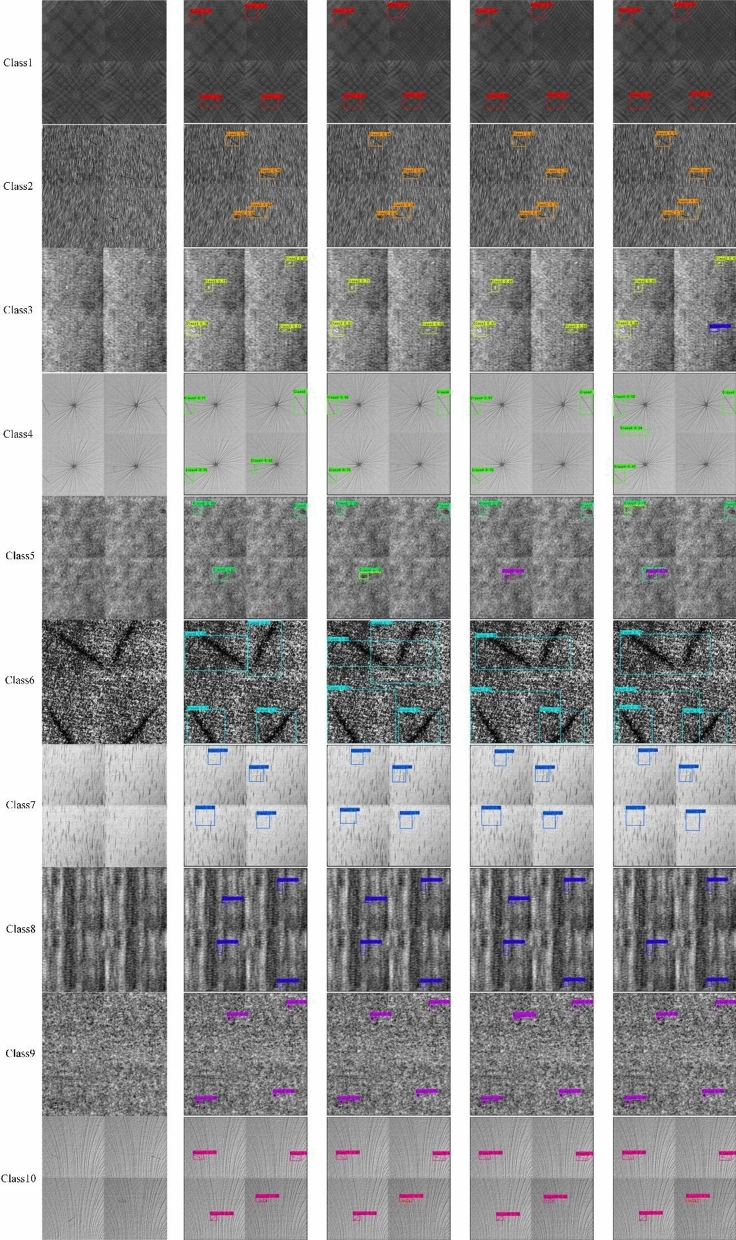
Comparison of single defect detection effects in fabric datasets.

Figure 15 shows the comparison of single defect detection effects in fabric datasets, from left to right, the original image, the proposed algorithm, Faster_RCNN, Yolo_v4 algorithms, and SSD. In order to fully demonstrate the defect detection ability of the algorithm proposed in this paper, each category in the figure is stitched together by four similar pictures, compared to directly entering a picture for detection, because the pictures of the input network will be processed into the size of 416 × 416, so the use of this stitched picture input network is equivalent to reducing the image resolution by 4 times, and the defects in the original picture will be directly reduced by 4 times, making the detection difficulty greatly increased, which also tests the generalization ability and feature mining ability of the algorithm. When evaluating the detection ability of the algorithm, the confidence threshold is generally set to 0.5, but the experimental verification finds that the SSD, Faster_RCNN, Yolo_v4 tiny and Yolo_v4 detection algorithms cannot be successfully detected under the threshold of 0.5, in order to make the comparison effect more obvious, the threshold of the algorithm in this paper is set to 0.5, and the threshold of the other comparison algorithms is set to 0.1. As can be seen from the figure, the proposed algorithm is better than the other four algorithms in 90% of the class detection results, and the confidence level is high. For class5 defects, because the defect is too small and similar to the background texture, the algorithm in this paper misses the detection of this type of defect, but the other four types of algorithms also have the same situation, and there is false detection. For class 6 defects, due to the fact that the left and right subgraphs of the stitched picture intersect with defects, the other four types of algorithms attribute the defects of the two subgraphs to the same detection box to varying degrees. This algorithm successfully separate the two kinds of defects, and position detection more accurate, showed the algorithm in this paper to a greater degree of refinement to detect defects, has a broad application prospect. Although Yolo_v4_tiny has a higher detection speed, the detection ability of low-resolution images is obviously insufficient, especially in the detection of defective samples of class 5, the algorithm has missed all the tests, and in other types of detection, the algorithm also has the problem of too low confidence, mainly because Yolo_v4 tiny only uses two feature layers for detection, so it appears to have insufficient performance in the detection of small samples.

### Multi-defect fusion detection

**Figure 16:**
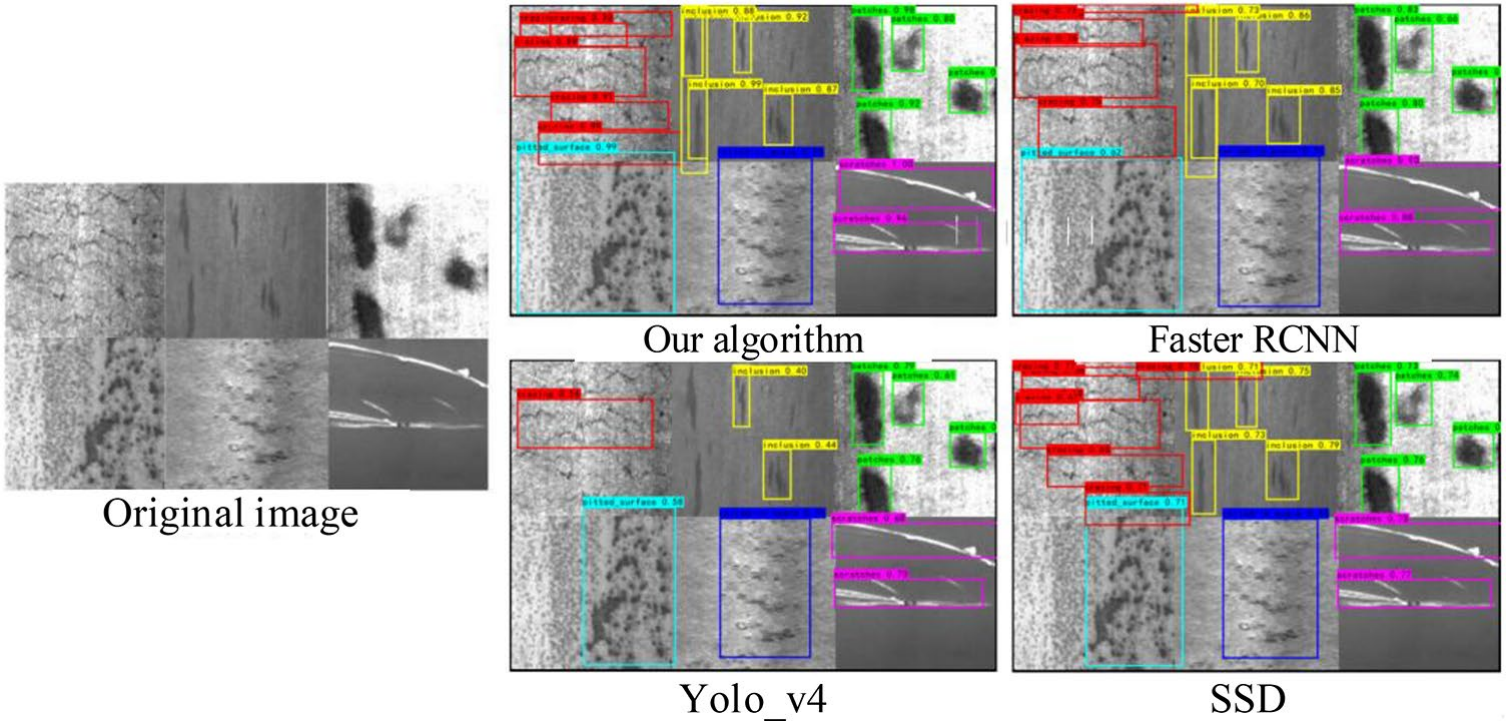
Fusion detection results of strip datasets.

**Figure 17:**
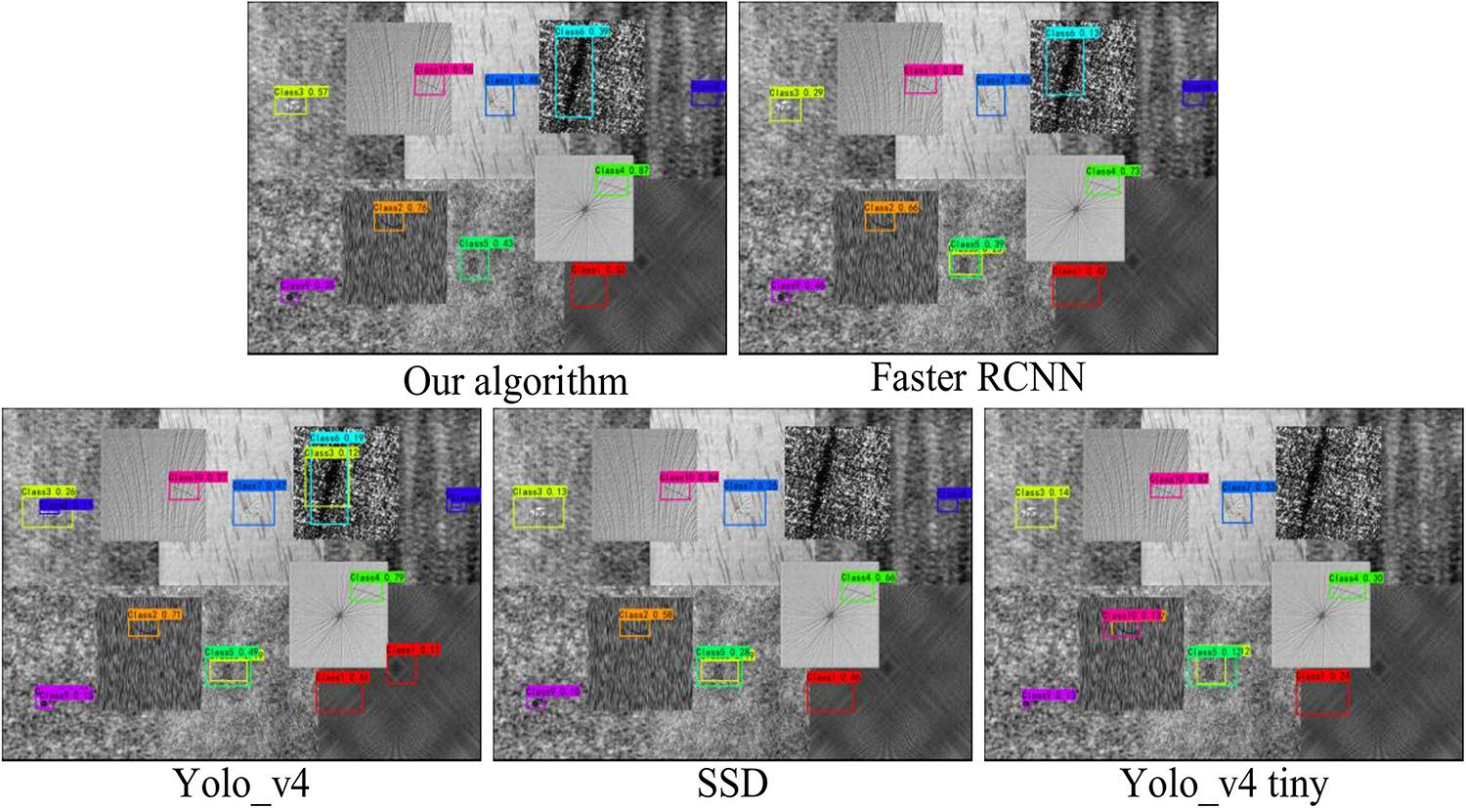
Fusion detection results fabric datasets.

In order to evaluate the ability of the algorithm to detect multiple defects at the same time, as shown in Figs. 16 and 17, all types of defects in the two data sets are fused into one picture and input various detection models to evaluate the detection ability of the model. For fused images, the resolution of each subgraph decreases exponentially, and the size of the defect appears smaller, making detection more difficult. To make the contrast stronger, the confidence threshold for all models is set to 0.1 in this experiment.

As shown in Fig. 16, for the strip datasets, the algorithm in this chapter can correctly identify all kinds of defects, and the confidence level is basically above 90%, while the unimproved Yolo_v4 have more missed detections and low confidence in the detection of crazing and inclusion. Although Faster RCNN and SSD algorithms can basically achieve all types of defect detection, their detection position is not accurate enough, and the confidence level is generally less than 0.7.

In order to evaluate the ability of the algorithm to detect multiple fabric defects at the same time, as shown in Fig. 17, all ten defect categories were fused into a single image and input into various detection models to evaluate the detection ability of the model. Since the images of the input model will be preprocessed to the size of 416 × 416 for detection, the resolution of each sub-image actually decreases by more than 6 times for the fused images, and the size of the defects will appear smaller, thus increasing the difficulty of detection. In order to make the comparison stronger, in this experiment, the confidence threshold of all models was set to 0.1.

The detection results can be seen that although the confidence value of the algorithm in this paper is lower than 0.5 in the detection of class 5, class 6, class 7 and class 9, it is basically maintained above 0.3, and ensures that all defect types are correctly identified and the defect location is accurately identified. In contrast, in other 4 types of comparison algorithms, the detection confidence of various types is below 0.1, and the position of the detection box is not precise enough; the 4 types of algorithms have misdetected the detection of the defective samples of class 5, and the detection of the 6th type of samples has also appeared to varying degrees of error detection and missed detection. Yolo_v4 treats the normal position as a defect in the detection of class 1 samples. yolov4_tiny missed the detection of the class 6 and class 8 samples. The comparison shows that the fabric surface defect detection algorithm in this paper can still maintain good detection performance in the case of low image resolution, and has strong anti-interference ability.

## Conclusion

In this paper, an improved Yolo_v4 based fabric surface defect detection algorithm is proposed. On the basis of Yolo_v4 algorithm, four main improvements are proposed in this paper: (1) An improved clustering algorithm is proposed to make Anchors more suitable for specific data sets by combining the advantages of K-Means and DBSCAN clustering algorithm; (2) The backbone feature extraction network of Yolo_v4 was modified to ECA-DenseNet-BC-121 to strengthen the feature extraction ability; (3) The five-fold convolution process in the PANet module was improved, and the DCFE module was proposed to replace it to further improve the model performance; (4) Use transfer learning to improve the problem of insufficient data. Experimental results show that the proposed algorithm is superior to SSD, Faster_RCNN, Yolo_v4 tiny and Yolo_v4 algorithms in fabric defect detection performance. Especially for low-resolution images, the proposed algorithm shows strong feature mining ability and can effectively detect small defects.

## Data Availability

Datasets generated and/or analyzed during the current study are available from the corresponding author upon reasonable request.

## Code availability

The code that support the findings of this study are available from the corresponding author upon reasonable request.
